# Effects of Silicone Breast Implants on Human Cell Types In Vitro: A Closer Look on Host and Implant

**DOI:** 10.1007/s00266-021-02762-x

**Published:** 2022-01-24

**Authors:** Maartje J. L. Colaris, Tim Ruhl, Justus P. Beier

**Affiliations:** Department of Plastic Surgery, Hand Surgery-Burn Center, University Hospital RWTH Aachen, Pauwelsstraße 30, 52074 Aachen, Germany

**Keywords:** Silicone breast implants, Breast implant illness, Silicone gel, Implant shell, Macrophages, Fibroblasts

## Abstract

**Background:**

Silicone (gel) breast implants (SBI) are used world-wide for breast augmentation, and reconstruction or to correct breast deformities. They consist of two compounds: an elastomer silicone shell (envelope) and a silicone gel filler (core). Breast Implant Illness (BII) is a term used for women with SBI, who suffer from various of symptoms including myalgia, arthralgia, fatigue, fever, dry eyes and/or dry mouth (sicca), as well as cognitive disturbances, which are rated by these woman as response to SBI. The pathogenesis of these adverse effects as well as the histocompatibility and the SBI-cell interaction of silicone and its surrounding tissue (implant-host tissue interface) is a subject of current research. The main purpose of this review is to provide an overview of the current knowledge regarding the effects of silicone (gel and elastomer surfaces) of a SBI on different human cell types from experimental - in vitro - models.

**Methods:**

A comprehensive research was conducted by two independent reviewers in March and July of 2020 in the PubMed, MEDLINE, and Cochrane databases.

**Results:**

A number of 1328 articles on this topic were initially identified, of which 62 could be finally included an analysed in this review.

**Conclusion:**

SBI may lead to a physiologic pro-inflammatory and foreign body host response with fibrous encapsulation accompanied by a disturbed Th17/Treg balance and IL-17 production. No causal relationship is known for systemic symptoms and/or autoimmune outcomes in the context of BII.

**Level of Evidence III:**

This journal requires that authors assign a level of evidence to each article. For a full description of these Evidence-Based Medicine ratings, please refer to the Table of Contents or the online Instructions to Authors www.springer.com/00266.

## Introduction

Silicone breast implants (SBI) were introduced first by Cronin and Gerrow in the 1960’s [1]. Initially, they were considered as moderate risk devices (Class II). In the early 1980’s, concerns arose about the safety of SBI resulting from local complications and adverse outcomes by new surveillance systems of The Food and drug administrations (FDA) [2]. The FDA reclassified SBI into higher-risk devices (Class III), including products that need Premarket approval (PMA), and asked manufacturers for providing data demonstrating safety of the devices [2].

In 1992, the FDA restricted the use of SBI based on inadequately addressed public concerns. This was accompanied with a call for studies on device performance and safety to improve surveillance of clinical outcomes [2, 3]. SBI were approved and re-introduced by the FDA in 2006 for the manufacturers Mentor and Allergan. However, because of limited data on long-term outcomes, the FDA required the manufacturers to conduct post-approval studies. These investigations could not find an association of SBI with Connective tissue diseases (CTD) or breast cancer [4, 5].

Since the first presentation and use for application, a controversy has arisen on the safety and adverse effects of SBI, especially, if SBI elicit inflammatory responses and/or autoimmune diseases/reactions. From the beginning on, SBI associated with certain clinical local side effects, such as pain, capsular contracture, implant rupture and silicone leakage. Many women still undergo a breast augmentation with SBI in aesthetic and reconstructive surgeries, *i.e.*, after mastectomy [6–8]. SBI are supposed to be associated with atypical systemic symptoms such as myalgia, arthralgia, fatigue, fever, dry eyes and/or dry mouth (sicca), as well as cognitive disturbances [9, 10], termed as the condition “Autoimmune/inflammatory syndrome induced by adjuvants” (ASIA) [11]. They were also supposed to be associated with an increased risk of developing inflammatory and autoimmune reactions [12, 13]. On the other hand, there is evidence for an increased incidence of a well-defined rare identity that can occur in SBI patients, *i.e.*, Breast implant related anaplastic large T cell lymphoma (BIA-ALCL), an uncommon form of non-Hodgkin lymphoma [14].

The controversy on SBI safety vs. adverse effects continues with the terminology of “Breast implant illness” (BII), which is subject of current research [15]. However, the effects of silicone on numerous physiological processes of the surrounding soft-tissue on the cellular level after systemic exposition (e.g. gel bleeding or implant rupture) have been hardly investigated. The main purpose of this review is to provide an overview of the current knowledge regarding the effects of silicone (gel and elastomer surfaces) of a SBI on different human cell types from experimental-*in vitro*-models. Before proceeding to the experimental part, in addition we firstly describe the clinical scenario and atypical systemic symptoms, followed by postulated pathophysiologic hypotheses, onto BII. Findings were summarized and presented in an Implant-Cell-Interaction diagram.

## Materials and methods

The foundation for this review was a systematic search and evaluation of the literature on the in vitro host-response on the SBI (silicone gel core and the elastomer shell), with the silicone in its original form. Furthermore, we describe the clinical scenario of the host with the incident of occurring systemic as well as local side effects after treatment of cosmetic and/or reconstructive SBI implantation.

A comprehensive search was conducted by two independent reviewers in March and July of 2020 using the following terms alone or in combination in the PubMed, MEDLINE, and Cochrane databases: silicone, silicone gel, silicone breast implant, silicone polymer, polydimethylsiloxane, PDMS, autoimmune inflammatory syndrome induced by adjuvants, ASIA, breast implant incompatibility syndrome, SIIS, breast prosthesis syndrome, breast implant illness, monocytes, macrophages, fibroblast, adipose tissue-derived stem cells, adipose stem cells, ADSC, ASC, adipocyte, breast epithelial cells, breast luminal epithelial cells, breast ductal epithelial cells. For describing the experimental part of this manuscript, we searched on different human cell types, which are in contact with an SBI within the human breast. These cell types were used in the search in combination with SBI/silicone terms.

All articles from the initial search were independently screened for eligibility based on title and abstract. Because one of the authors is fluent in Dutch and German, filters were set to include all articles in English, Dutch, and German. Animal studies, observational studies, case-control studies, randomized controlled trials, meta-analysis and reviews were included. Case reports/series, conference abstracts, commentaries and letters to the editor were excluded. Studies on processed silicones respectively silicone gel not in its original form from a SBI as well as silicone fluids, e.g. silicone oil, were also excluded.

A formal statistical analysis of the eligible studies was not performed because of the methodologic and clinical heterogeneity. A detailed systematic review of the diverse outcomes was undertaken instead.

## Results

**Fig. 1:**
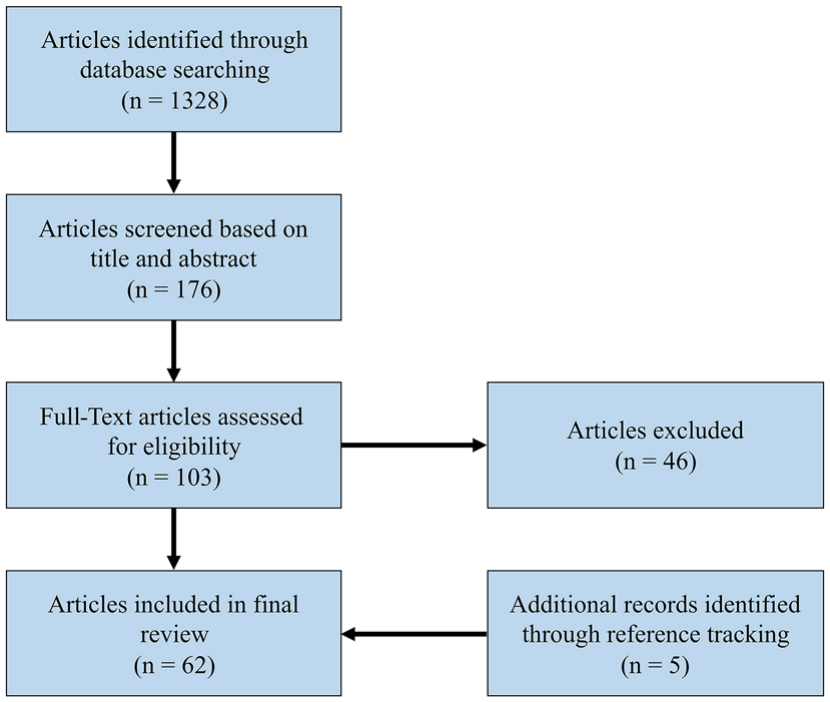
PRISMA flow diagram of literature research

The primary search yielded 1328 articles. The titles of remaining articles were screened for relevance, after which 176 abstracts were reviewed according to our inclusion criteria. The remaining 103 articles were read in their entirety and their references scoured for articles that escaped our primary search criteria. Of these, 46 were excluded based on predetermined criteria. The remaining 57, along with 5 articles that were discovered by reviewing of references, resulting in a total of 62 articles, were included in this review (Figure 1).

### Clinical scenario and systemic symptoms among BII patients

In the past sixty years, the character of SBI-related complaints did not change, although their names were adjusted [6]. A variety of terms have been applied to the clinical spectrum of SBI patient concerns, e.g. ‘adjuvant breast disease’ and ‘human adjuvant disease’, ‘Autoimmune/inflammatory syndrome induced by adjuvants (ASIA)’ introduced by Shoenfeld in 2011 [11]. This has been further specified as ‘Silicone implant incompatibility syndrome’ (SIIS), or the more well-known name ‘Breast Implant Illness’ nowadays [15, 16].

The atypical clinical spectrum of health complaints that SBI patients report, are myalgia, myositis or muscle weakness, arthralgia and/or arthritis, chronic fatigue, unrefreshing sleep or sleep disturbances, cognitive impairment (concentration problems, memory loss), pyrexia, sicca as also neurological manifestations (e.g. TIA, white matter lesions) are described [11]. Moreover, several other symptoms are frequently present, such as Raynaud’s phenomenon, recurrent respiratory tract infections, recurrent cystitis, livedo reticularis, headache, alopecia or hair loss, skin abnormalities, gastrointestinal symptoms (irritable bowel syndrome), night sweats and lymphadenopathy, fibromyalgia (FM), Chronic fatigue syndrome (CFS) and allergies. These clinical findings in patients with SBI mimic the clinical picture of FM, which is why it has been postulated that BII is not a new disease [17].

**Fig. 2:**
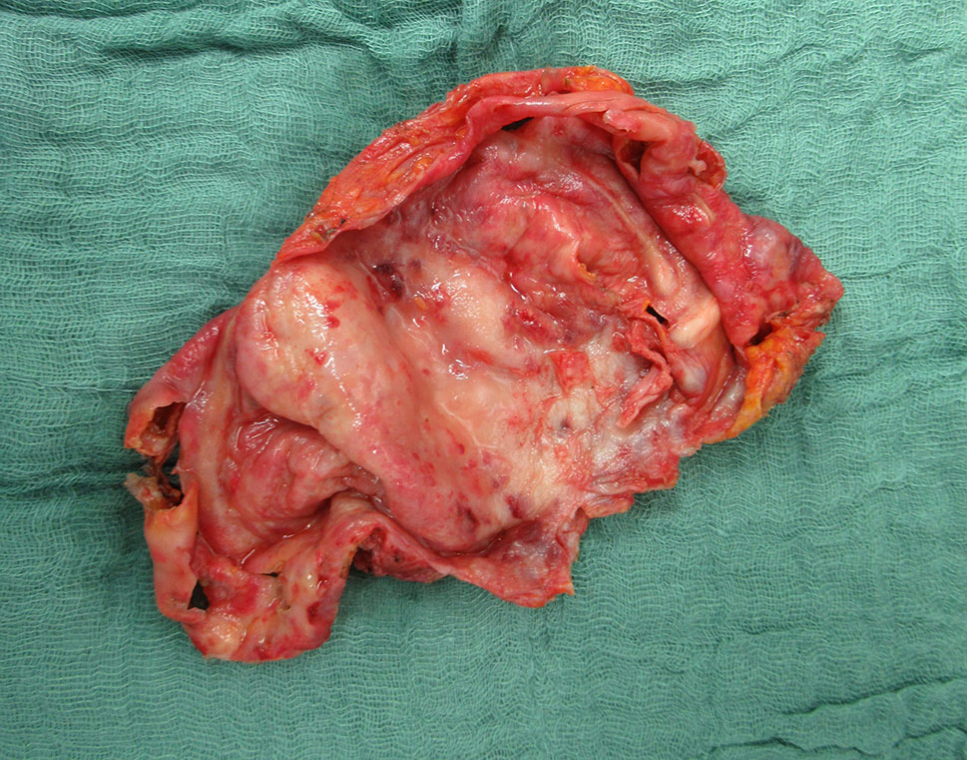
Capsular fibrosis (macroscopically): intraoperative macroscopic appearance of the inner aspect of the capsular fibroses after excision in a patient with previous implant-based breast reconstruction (followed by radiation therapy) for breast cancer

Autoimmune diseases that occur in SBI patients are Rheumatoid arthritis (RA), Sjogren’s syndrome and other Connective tissue diseases (CTD), vasculitis, granulomatous disease and others like multiple sclerosis and Hashimoto’s thyroiditis. Well-known local complications of SBI are capsular contracture (Figure 2), implant rupture, breast pain, asymmetry and infection [18]. Furthermore, an increased occurrence of a deficient humoral immune system is reported [19]. Vitamin D may act as a regulatory agent of the immune system. Vitamin D deficiency is found to be related to the presence of auto-antibodies in patients with silicone implant incompatibility syndrome [20]. However, whether Vitamin D deficiency is also related to the presence of autoantibodies in SBI patients without complaints as well as healthy women remains unknown by the lack of a control group the mentioned study. Interestingly, it is still controversial whether SBI increase the risk of autoimmune disorders [21]. Existing evidence on pathophysiological mechanisms concerning the local and systemically adverse effects is limited.

Coroneos et al. have published the largest prospective epidemiologic study of patient safety and implant-specific outcomes for SBI in 2019 [12]. 99,993 patients of the FDA LPAS database followed up for 10 years by two SBI manufacturers: Allergan and Mentor Corp (Mentor). In this study, SBI were associated with higher rates of Sjogren’s syndrome (SIR 8.14), scleroderma (SIR 7.00), rheumatoid arthritis (SIR 5.96), stillbirth (SIR 4.50), and melanoma (SIR 3.71). Furthermore, SBI are associated with decreased rates of fibromyalgia and lung cancer compared with the general population [12]. All reported events represented new diagnoses compared to the patient’s baseline [12]. However, the associations reported herein are inconclusive, given the limitation of missing data from individual patient-level analyses. Also, no associations with brain cancer or suicide were found [22]. The large meta-analysis by Balk et al., including 32 observational studies, concluded that there is no evidence for an association between SBI and any health outcomes [4]. However, the authors observed a decreased risk for breast and endometrial cancer, and increased risk for lung cancer, rheumatoid arthritis, Sjogren’s syndrome, and Raynaud syndrome [4]. Limitations of this study were a general lack of adequate accounting for possible confounders (e.g. studies not specific to SBI) [4]. The most common risk of bias among studies was that analyses were mostly inadequately adjusted (4 studies) or not adjusted (27 studies) for potential confounders; several studies reported adjusted analyses for some outcomes, but unadjusted analyses for other outcomes [4]. There was no association between higher rates of suicide among SBI patients in comparison to the general population [23]. Others reported on an association between SBI and autoimmune/rheumatic disorders with an OR of 1.22 (95% CI 1.18–1.26) [13]. This study is however also not fully unbiased. The Breast Implant Follow-Up Study, a large multicenter observational study, based on five-year safety data of the FDA LPAS database for more than 55,000 subjects showed that Natrelle round SBI do not increase the risk of any systemic disease over expected rates based on national norms or when compared with saline implant outcomes, regardless of the indication for implantation [5]. Also, the risk for any cancer diagnosis was not elevated.

The rarity of nonspecific symptoms allocated by SBI patients, known as BII, prevents an adequate duration of follow-up time. The FDA concluded that a study would need to collect data on hundreds of thousands of women for more than 10 years to confirm an association [2]. Knowledge about breast implant prevalence is essential for assessing the absolute risk and public health impact of breast implant-related health problems. The estimated national Dutch prevalence of breast implants among women between 20 and 70 years is 3.0%, ranging from 1.7% at 21 to 30 years to 3.9% between 51 and 60 years (sensitivity 79.9%; specificity 99.2%) [9]. In a descriptive cohort study of 80 women with SBI and unexplained systemic symptoms, 75% of the women reported pre-existent allergy prior to implantation [24]. The majority of women received SBI for cosmetic reasons [6, 24]. The median age at time of diagnosis is around 48 years (range 22–78 years) [6, 24]. The median total exposure time to SBI was 14.5 years (range 2–42 years) [24]. The development of the symptoms after implantation of SBI begins after a symptom-free period of years with a median of 4.5 years (range 1 month–30 years) [6, 24]. While being exposed to SBI, approximately 14% of patients developed a confirmed autoimmune disease at a median time of seven years after first implantation (range 3–30 years) [6, 24]. A more recent study about health complaints in SBI patients showed that the adjusted prevalence of self-reported health complaints related to BII was not higher in women with SBIs than in women without breast implants [7]. However, It has also been shown that no differences in the prevalence of BII exist in dependence of the implant indication (cosmetic vs. reconstructive) [7]. FM and CFS were more common in women with SBIs compared to controls, and the presence of a chronic disease was found to be an independent predictor for the development of BII [7].

After explantation of the SBI, complaints were reduced in 75% of the patients, whereas in patients with autoimmune diseases, improvement only occurred when explantation was combined with immunosuppressive therapy [25]. Women with SBI and autoimmune diseases have shown differences in Human leukocyte antigen (HLA) typing as compared with asymptomatic patients [26]. Beside the genetic predisposition of autoimmune diseases, (a history of) allergic reactions could also act as an inductor of systemic autoimmune reactions [27].

### Pathophysiologic hypotheses of BII

Different hypotheses have been postulated for the development of unexplained systemically symptoms in SBI patients: silicone leakage, also known as silicone ‘bleeding’ and/or rupture of the implant, silicon toxicity and the SBI functioning as a nociceptive stimulator [6, 28, 29]. Also, many SBI patients share experience, seek support, and express frustration on social media with discussions about BII, which may have a contributable influence on the concerns that these patients are sharing with each other [30–32]. Stress and other cognitive and emotional mechanisms may affect many physical symptoms and sensations as well [32]. Concerning the local implant-host tissue interface, BII symptoms do not correlate with any particular implant type, surface or fill [33]. The biofilm hypothesis declares that chronic infection was found in 36% of symptomatic BII patients, with P. acnes being the most commonly found organism [33]. In addition, symptomatic BII patients had an increased incidence of capsule synoviocyte metaplasia than a matched cohort that did not have BII symptoms [33].

In previous studies, it has been postulated that implant rupture and/or ageing can be important factors for eliciting an inflammatory response or for triggering the immune system upon silicone particles migrating throughout the body [28]. The phenomenon of gel bleed is known for all types of SBI [34–36]. The migration of the silicone gel particles throughout the body is accompanied by lymph node and thoracic silicone infiltration, with giant-cell granulomas and small silicone vacuoles found in lymph node biopsies [28, 37]. Droplets and plaques containing silicone are found in tissue samples of different parts of the brain as well as in the spinal cord [36]. Silicon-containing particles are transported to the regional lymph nodes, possibly resulting in an adjuvant effect [38]. The amount and size of the silicone molecules may determine the induction of the apoptotic processes by silicones, known as ‘silicon toxicity’ [29]. Exposure of cultured human Jurkat cells, a human T lymphoblast non-adhering cell line, to low molecular weight methylcyclosiloxanes, the smallest cyclic silicone oligomer octamethylcyclotetrasiloxane (D4) and the decamethylcyclopentasiloxane (D5), can induce cell death by apoptotic processes such as cleavage of caspase substrates and DNA fragmentation [29]. Also, the cell sensitivity to the toxic silicone compounds seems to differ between cell types [29]. The use of silicone in the environment and many other medical devices brings silicone also into the body of women without SBI. Peters et al. demonstrated consistent levels of silicon in the blood and plasma of control women without exposure to SBI [39], while serum silicone levels were elevated in women with SBI [40, 41]. The clinical relevance of these single studies is questionable regarding their pathophysiological consequences in SBI patients.

### Experimental models on different cellular phenotypes upon silicone exposure

Until now, there is still conflicting evidence about the supposed histocompatibility and the material-cell interactions of silicone and its environment after implantation in the human body. When considering body or tissue responses upon silicone contact, the relevant cell types and phenotypes have to be identified.

Most *in vitro* investigations targeted on immune cells, especially the reactions of macrophages and their progenies monocytes, have been measured when the cells were stimulated with silicone gel or different silicone elastomer surfaces. Other cells that have been exposed to silicone *in vitro* were Human fibroblasts and umbilical vein endothelial cells (HUVEC). No *in vitro* or *in vivo* studies have been found for the interaction of Adipose-derived stem cells (ADSCs) and/or adipocytes and breast epithelial cells with silicone gel or silicone surfaces.

#### Monocytes/Macrophages (M1) upon silicone exposure

Macrophages could mediate silicone-induced adverse responses, such as Foreign body reaction (FBR) and fibrous encapsulation [42]. Macrophages are the key cells forming foreign body giant cells [43]. The foreign body reaction can be divided into different phases: (1) after implantation, the biomaterial is coated in a protein-layer of the surrounding wound fluid and neutrophils reach the wound site; (2) monocytes differentiate into macrophages which develop into foreign body giant cells and cause the recruitment of fibroblasts; (3) fibroblasts begin to isolate the implant from the surrounding tissue by depositing collagen and the fibrous capsule [44]. Implantation of a medical device or biomaterial into the human body in general leads to a FBR, marked by different phases: protein adsorption on the implant surface, monocyte/macrophage adhesion, acute inflammation, chronic inflammation, foreign body giant cell formation out of macrophages, fibroblast activation and fibrous capsule formation [45]. The proteins that adsorb onto the implant surface determine cell adhesion to the biomaterial [45]. The degradation of the biomaterial depends onto the chemistry of the biomaterial surface [45]. The host response to SBI as a biomaterial differs from the general FBR by the production of the cytokine IL-17 following exposure to silicon-containing particles after apoptosis by macrophages [38]. This induces an invagination of neutrophils that are activated and produce Reactive oxygen species (ROS) and release enzymes such as myeloperoxidase [38]. Following acute inflammation, chronic inflammation is identified by the presence of mononuclear cells, i.e., monocytes and lymphocytes, at the implant site [45]. Tavazzani et al. investigated the *in vitro* interaction between silicone gel and monocyte-macrophages by harvesting and culturing human peripheral blood monocytes with silicone gel droplets (<8 μm) embedded in a type I collagen matrix [43]. The histological evaluation indicated phagocytosis of the silicone gel within hours of exposure to the material and the silicone-exposed cells appeared to be larger and more granular when compared to controls [43]. Furthermore, silicone-exposed cells formed spindle-shape phenotypes and multinucleated cells, which were not detected in the collagen-controlled cultures [43]. There was no evidence of cytotoxicity after silicone phagocytosis within the incubation time for up to 7 days (> 95% cell viability at 24 h). The results of the cytokine analysis showed an increased secretion of IL-1 by M1 macrophages upon exposure to silicone gel at 24 h (*p* < 0.01) [43]. There were no effects on the release of TNF-α or IL-6. IL-2 dependent cytotoxic T cells (CTLL) show no difference in activation after exposure to silicone gel [43]. Rhie et al. assessed the functional changes of macrophages and lymphocytes in a series of immunotoxicologic assays after *in vitro* cultivation of the cells with silicone gel [46]. However, in contrast to Tavazzani et al. they found that direct contact of macrophages with silicone gel is a primary cause of acute immune activation that might be related to foreign body reactions. After 3 days incubation, the silicone cytotoxicity on macrophages was determined using Yac-1 cells as target cells (ratio of target cells to macrophages 1:5). Silicone caused a higher functional cytotoxic activation of macrophages to target cells incubated in silicone plates compared to macrophages cultivated on normal, conventional plates (65.2% vs. 19%; *p* < 0.01) [46]. Furthermore, the primary T-dependent immunoglobulin M antibody response, in which macrophages involve as antigen-presenting cells, is also affected by silicone gel [46]. Because primary T-dependent immunoglobulin M response is mediated by B- and T-lymphocytes along with macrophages, the authors also investigated the effect of silicone gel on lymphocytes to ascertain whether only macrophages play a role in silicone gel-mediated stimulation of the antibody formation (T-dependent immunoglobulin M) [46]. They proved that B- and T-lymphocytes are not directly affected by silicone gel, so that the stimulated T-dependent antibody response could be primarily driven by macrophages.

Naim et al. investigated the activation of monocytes/macrophages by silicone elastomers, silicone gels and oils, that were pre-adsorbed with various plasma proteins via measuring the cytokine release [47]. They showed that plasma proteins (albumin, fibrinogen or IgG), adsorbed to very hydrophobic surfaces, increase the monocytes secretion of IL-1β, IL-6 and TNF-α [47]. The difference in monocyte activation cultured on either silicones or Tissue culture grade polystyrene (TPST) is not influenced by the quantity of protein adsorption. The silicone gel, silicone oil and silicone gel/oil combination causes the monocytes to secrete nearly twice the amount of all above described cytokines in comparison with the silicone elastomer. The silicone elastomer is very hydrophobic. Hydrophobic surfaces are more denaturing to adsorbed proteins than hydrophilic surfaces [47]. Plasma proteins, when adsorbed to a hydrophobic surface, become denatured and cause monocytes to secrete pro-inflammatory cytokines, IL-1β, IL-6, and TNF-α by an unknown mechanism [47].

#### Fibrous encapsulation of SBI

The formation of Fibrous capsular contractures (FCC) is a well-known local reaction onto SBI. The response of the surrounding tissue to a SBI, the foreign body reaction, is the basis for FCC. However, the pathogenesis of FCC on the cellular level is a subject of investigation.

McCauley et al. examined the responses of Human dermal fibroblasts (HDFs) exposed to silicone polymers (silicone gel and elastomer envelope) of SBI [48]. HDFs underlie reduced viability when being co-cultured with silicone gel as well as with the silicone envelope. HDFs show no proliferation during 7 days of culture on the elastomer envelope, while cell growth increases mildly at exposition to silicone gel after 7 days. The inhibition of fibroblast proliferation on silicone gel as well as on the elastomer envelope correlates with the low number of fibroblasts seen in the fibrous capsule histology [48]. Furthermore, HDFs are characterized by markedly changed morphology, with a more oval and colony formatted growth pattern as well as a twofold increase in the rough endoplasmic reticulum, when they are stimulated with the silicone gel compared to stimulation with the silicone envelope [48].

The host-response on the SBI surface also depends on the surface-structure. The elastomeric shell of a SBI is either ,smooth’ or ,textured’. Seyhan et al. have investigated differences in the response of fibroblasts on the different surfaces of SBI [49]. After a 4-week incubation period, fibroblast proliferation on textured surfaces is at 20% when compared to fibroblast proliferation on smooth surfaces. TGF-β1 production is lowered by smooth surface fibroblasts compared to textured surface cells. These findings indicate that the proliferative properties of human fibroblasts are influenced by the different surfaces of SBI *in vitro* linked to the TGF-β pathway. Kyle et al. investigated human Breast derived fibroblasts (BDFs) cultured on PDMS surfaces and compared them to commercially available smooth and textured SBI surfaces [50]. Cell attachment and proliferation, cell apoptosis as well as pro-inflammatory and pro-fibrotic cytokine secretion by BDFs were investigated. BDFs attach stronger onto ADM PDMS surfaces than to both smooth and textured implant surfaces. Whereas, there is no effect by smooth and textured implant surfaces on the cell attachment. There is a increase in BDF cell proliferation on ADM PDMS surfaces when compared to smooth and textured implant surfaces after 24 h [56]. After 1 week, BDFs show stronger proliferation on smooth than on textured implant surfaces [50]. The expression of IL-8 is down-regulated in BDFs on ADM PDMS. In addition, textured implant surfaces increase cell apoptosis and down-regulates IL-8 release in BDFs when compared to cells on smooth implant surfaces [50]. After 48 h, TNF-α is down-regulated in BDFs on ADM PDMS surfaces in comparison to both smooth and textured SBI surfaces. The same applies for TGF-β1, which is found reduced after one week, whereas its level is higher on smooth than on textured surfaces [50]. There is no difference in expression of collagen type 1 on any of the silicone surfaces.

The secretion of pro-inflammatory/ pro-fibrotic cytokines IL8, TNF-α and TGF-β are all significantly up-regulated in contracted fibrotic breast capsules around SBI [51]. The immunopathogenesis in the development of SBI elastomer capsule contracture could be explained as follows: Monocytes and macrophages are activated by the silicone of SBI, which induces a pro-inflammatory immune response resulting in the secretion of IL8 and TNF-α. This in turn leads to migration of more monocytes with further up-regulation of IL-8 and TNF-α, with the consequence of a chronic inflammation. This is accompanied by differentiation of fibroblasts into myofibroblasts mediated by the profibrotic cytokine TGF-β [52]. IL-6, has an additional profibrotic role in activating fibroblast to myofibroblast transition [53].

**Fig 3:**
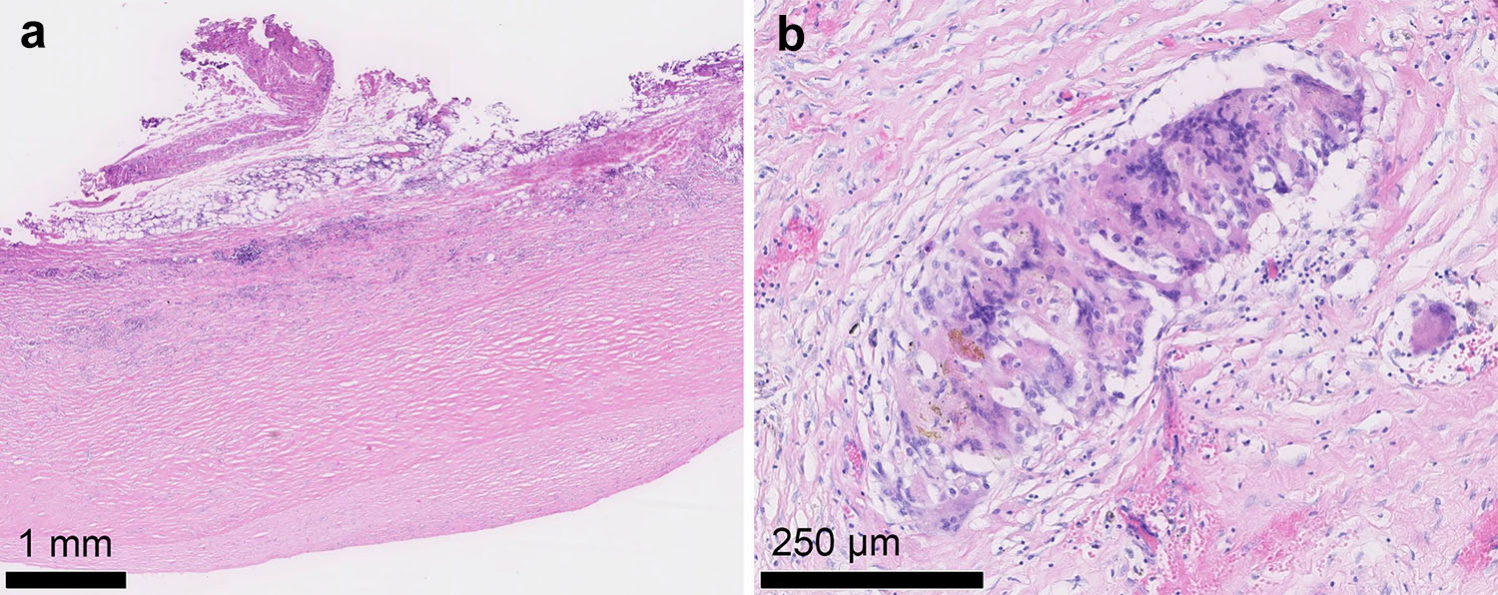
Histological aspect of capsular fibrosis (same patient as Figure 2): **a** hematoxylin and eosin (H&E) staining of capsular fibrosis tissue, overview of horizontal section through capsular fibrosis tissue with signs of chronic inflammation **b** higher magnification showing foreign body reaction with accumulated giant cells

**Fig 4:**
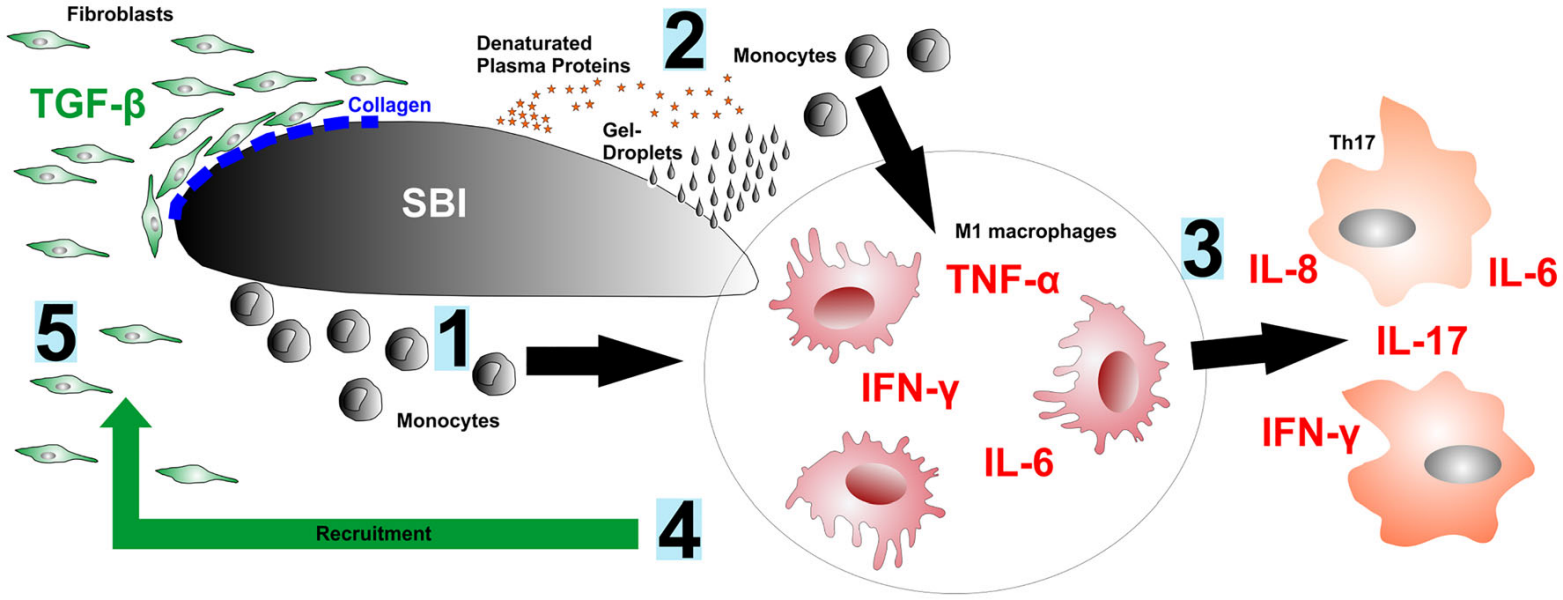
Implant-Cell-Interaction diagram: [1] monocytes differentiate into M1 macrophages after contact to SBI, perform silicone phagocytosis and secrete pro-inflammatory cytokines (TNF-α, IFN-γ, IL-6), [2] Denatured plasma proteins cause monocytes to secrete pro-inflammatory cytokines [3] IL-6 is produced during the inflammatory response and induces the development of Th17 cells with the leading cytokines IL-8, IL-17, IL-6 and IFN-γ. [4] M1 Macrophages develop into foreign body giant cells and cause the recruitment of fibroblasts. [5] fibroblast isolate the SBI from the surrounding tissue by depositing collagen and the fibrous capsule mediated by the profibrotic cytokine TGF-β

Furthermore, capsules around SBIs contain inflammatory cells (Figure 3a, b) that are predominantly Th1/Th17 cells, releasing high amounts of IL-6, IL-8, IL-17 and IFN-γ as well as defective regulatory T-cells, which possibly may result in the development of inflammatory/autoimmune diseases [54]. The disturbed Th17/Treg balance leads to a malfunction of the local T-cells with the result of increased production of profibrotic cytokines [54]. IL-1β and TNF-α are enhanced by IL-17 [54]. IL-1β has a regulatory role in fibroblast growth, proliferation and protein synthesis [43]. The pathogenesis of capsular contracture appears to be multifactorial. Capsular contraction is associated with increased number of Circulating immune complexes (CIC) as well as other serum parameters like procollagen III (a marker of active fibrosis), Anti-polymer antibodies (APA) and Soluble intercellular adhesion molecule-1 (sICAM-1) [55]. Furthermore, according to a number of studies, capsular contraction is frequently associated with increased serum hyaluronan levels in SBI patients as compared to healthy SBI patients [56–58]. Propionibacterium acnes infection of the breast implant shell and capsule is postulated as disease model for capsular contracture [59, 60]. Another immunological and bacterial factor involved in the development of capsular contracture is the presence of staphylococcus epidermis leading to the formation of periprosthetic inflammation [52, 54]. IL-6 is produced during the inflammatory response, which inhibits the generation of T-cells and induces the development of Th17 cells by converting naïve CD4^+^ cells (for overview see Figure 4) [44].

Another central topic in cell-biomaterial interaction research is cell adhesion and cell spreading on the biomaterials. Fibroblast attachment decreases depending on implant surfaces properties (smooth and textured) compared to a foam polyurethane surface as well as tissue culture plastic controls [61]. Fibroblast proliferation is significantly decreased on foam polyurethane as well as on textured implant surfaces, but not on smooth implant surfaces. This stands in contrast to Human umbilical vein endothelial cells (HUVEC) proliferation. The formation of focal adhesions and fibronectin fibrillar structures by human fibroblasts to fibronectin-precoated smooth and textured SBI has been investigated by van Kooten et al. [62]. Cell adhesion starts off with formation of focal contacts, followed by fibronectin-containing matrix assembly. Fibronectin production and assembly as well as the orientation of fibronectin fibrils and focal contacts are related to the underlying grooved surface, in which fibronectin formation is guided by the grooves or groove walls [62]. Cells do not proliferate on silicone surfaces without fibronectin predisposition. Human fibroblasts, produce up to 3.5-fold as much fibronectin mRNA on textured SBI surfaces when compared to smooth surfaces [62]. Cells do not grow to confluence on non-coated silicone surfaces.

## Conclusions

This systematic review evaluated the variety of local and systemic host reactions to SBI, especially if these reactions represent a rather nonspecific inflammatory and/or a specific immune-mediated reaction. Data on an association between SBI and inflammatory/autoimmune outcomes have been debated for decades. No causality could be found in the most recent systematic review [4]. Authors concluded that the evidence is very weak for associations specific to women with silicone gel implants. Few comparative studies had to be restricted to this implant type and most studies did combine analyses with saline-filled implants. Furthermore, included studies were inconsistent, differences between the studies in how the data were analyzed made comparison difficult and studies were rarely adequate adjusted for potential confounders. However, the existence of an association between health complaints and SBI has been less investigated. Most studies focused on disease outcomes rather than health symptoms. Moreover, data from large epidemiologic studies on SBI patients with systemic complaints in relation to the entire SBI population are lacking. On the contrary, an association has not been ruled out to date. Remarkably, a comparative study on the prevalence of health complaints in SBI patients showed that health complaints were only higher in a group of self-reported women who made their complaints public in an online platform compared to controls.^10^ The adjusted prevalence of self-reported health complaints were not found to be significantly higher in SBI patients who did not made their complaints public in comparison to control patients without SBI.^10^ Women who reported their complaints online (e.g. social media and internet worrying patients) are expected to have the most severe complaints and thus do not constitute a representative control group for the entire SBI population due to selection bias. Even the largest epidemiologic studies of breast implant outcomes did not reveal a direct causality [5, 12]. Authors reported that baseline characteristics and comorbidities of patients were missing, which is especially important when adjusting analyses for known covariates or potential confounders among rare disease symptoms. To better understand the not-well defined phenomenon of BII, large prospective epidemiologic studies with adequate control groups (e.g. data from (inter)national breast implant registries) are needed to demonstrate any existing causality. After taking a close look on the clinical scenario as well as data from *in vitro* models onto SBI as a biomaterial, it appears that biomaterial-host’s cell interactions matches with the FBR phases following implantation of other medical devices or biomaterials. The host response to SBI as a biomaterial differs from the general FRB by the production of the cytokine IL-17 following exposure to silicon-containing particles after apoptosis by macrophages as well as the inflammatory Th1/Th17 cells predominantly found in the SBI capsule, which may result in the development of inflammatory/autoimmune diseases [38, 54]. We are aware that the design of our study may have several limitations: (A) This review does not qualify for a systemic review according to the definition of the Cochrane Handbook, which would have a greater number and quality of studies. Clinical studies on breast implant illness have their limitations concerning under powering, inadequately adjusting or not-adjusting for confounders, patient-reported symptoms which were not confirmed by a physician, lack on clarity of the existence of symptoms before the placement of breast implants and studies that failed an adequate control group. Especially, SBI patients reporting their medical complaints, are a selected group of patients, which differ from SBI patients without BII, leading to selection bias. (B) the available *in vitro* studies and evidences on the interactions of silicone and different human cell phenotypes remains limited, particularly regarding the interaction of silicone gel of the core and the silicone elastomer shell with its biological vicinity. A restriction for experimental research is to develop an environment that sufficiently simulates the *in vivo* situation of the silicone breast prosthesis. *In vitro* models are an oversimplification of *in vivo* environmental conditions and the physical property of silicone gel (e.g. hydrophobicity, lower density, stickiness) restricts the possibility of an transfer to experimental culture conditions. (C) The differences of implant surfaces (e.g. texturing, size, shape), the production process of the implant, and the droplet size of the silicone gel are other variables that have to be considered, when trying to identify a stimulator for pathological outcomes, especially for an existing inflammatory and/or immune response. In conclusion, SBI (gel and/or shell) lead to a physiologic pro-inflammatory and foreign body host response with fibrous encapsulation accompanied by a disturbed Th17/Treg balance and IL-17 production. Locally as well as systemically symptoms, possibly as an adjuvant effect of migrating and accumulating silicone particles into lymph nodes and other body regions, can occur in a subgroup of SBI patients due to an until now unknown pathophysiologic mechanism. Further experimental-*in vitro*-and later on appropriate cocultured-*in vivo*-research is needed on how the immune system interacts acts as a host response at the human cellular level with SBI as a biomaterial.
